# Supplementary material for: Association of METS-IR index with Type 2 Diabetes: A cross-sectional analysis of national health and nutrition examination survey data from 2009 to 2018
Source: PLoS One. 2024 Nov 8;19(11):e0308597. doi: 10.1371/journal.pone.0308597 (PMC11548740; doi:10.1371/journal.pone.0308597)
Supplement: Attachment — Submitted filename: Response to Reviewers.docx [file pone.0308597.s001.docx]

Dear Editor,

I hope this message finds you well.

Thank you for your feedback on our manuscript titled "Association of METS-IR Index with Type 2 Diabetes: A Cross-sectional Analysis of National Health and Nutrition Examination Survey Data from 2009 to 2018" We are in the process of revising the manuscript as per your suggestions.

We hope that the revised version of the manuscript is now acceptable for publication in your journal.

Thank you for your time and consideration. We look forward to your confirmation and further instructions.

Best regards,

HOU

We would like to express our sincere thanks to the reviewers for the constructive and positive comments.

**Specific Comments**

**Comment 1: Please ensure that your manuscript meets PLOS ONE's style requirements, including those for file naming.**

**Answer:** Thank you for your reminder. We will carefully review and ensure that our manuscript adheres to PLOS ONE's style requirements, including the proper file naming conventions.

We appreciate your guidance and will make the necessary adjustments.

**Comment 2:** PLOS requires an ORCID iD for the corresponding author in Editorial Manager on papers submitted after December 6th, 2016. Please ensure that you have an ORCID iD and that it is validated in Editorial Manager. To do this, go to ‘Update my Information’ (in the upper left-hand corner of the main menu), and click on the Fetch/Validate link next to the ORCID field. This will take you to the ORCID site and allow you to create a new iD or authenticate a pre-existing iD in Editorial Manager.

**Answer:** Thank you for the reminder. I confirm that I have an ORCID iD, and I will ensure that it is properly validated in the Editorial Manager by following the provided instructions.

I appreciate your guidance on this matter.

**Comment 3:** Your ethics statement should only appear in the Methods section of your manuscript. If your ethics statement is written in any section besides the Methods, please move it to the Methods section and delete it from any other section. Please ensure that your ethics statement is included in your manuscript, as the ethics statement entered into the online submission form will not be published alongside your manuscript.

**Answer:** Thank you for your comment. We will move the ethics statement to the Methods section of the manuscript and ensure it is only included there. We will also make sure that the ethics statement is properly incorporated into the manuscript, as per the guidelines.

We appreciate your guidance on this matter.

**Comment 4:** Please review your reference list to ensure that it is complete and correct. If you have cited papers that have been retracted, please include the rationale for doing so in the manuscript text, or remove these references and replace them with relevant current references. Any changes to the reference list should be mentioned in the rebuttal letter that accompanies your revised manuscript. If you need to cite a retracted article, indicate the article’s retracted status in the References list and also include a citation and full reference for the retraction notice.

**Answer:** Thank you for your helpful comment. We will thoroughly review our reference list to ensure it is complete and correct. If any retracted papers have been cited, we will either remove them and replace them with relevant current references, or provide a rationale for their inclusion in the manuscript. We will detail any changes made to the reference list in the rebuttal letter that accompanies the revised manuscript.

Original references: [18]	2. Classification and Diagnosis of Diabetes: Standards of Medical Care in Diabetes-2021 [J]. Diabetes Care, 2021, 44(Suppl 1): S15-s33.

modified references: [18]	American Diabetes Association. 2. Classification and Diagnosis of Diabetes: Standards of Medical Care in Diabetes-2021. Diabetes Care. 2021 Jan;44(Suppl 1):S15-S33.

The original references were imported via EndNote, and the information regarding the authoring institutions was missing. After modification, this information has been added.

**Comment 5:** In the Ethics Statement section, the author should specify whether the study was conducted in accordance with the `Helsinki Declaration`.

**Answer:** Thank you for your valuable suggestions on our manuscript. We confirm that this study was conducted in strict accordance with the Declaration of Helsinki. In the upcoming revisions, we will explicitly mention this in the Ethics Statement section to ensure compliance and transparency.

**Comment 6:** The term "METS-IR" should be written out in full the first time it appears, as should "NHANES."

**Answer:** Thank you for your insightful comment. We will ensure that the term "METS-IR" is written out in full as "Metabolic Score for Insulin Resistance" upon its first mention, as well as "NHANES" as "National Health and Nutrition Examination Survey."
